# Progressive Liver Metastasis of Breast Cancer on Endocrine Therapy Showing Favorable Response to Additive Alternate-Day S-1 Therapy: A Case Report

**DOI:** 10.7759/cureus.115687

**Published:** 2026-09-02

**Authors:** Kasumi Arizumi, Shoji Oura

**Affiliations:** 1 Department of Surgery, Kishiwada Tokushukai Hospital, Kishiwada, JPN

**Keywords:** alternate-day s-1 therapy, breast cancer, discordant response, liver metastasis, luminal type

## Abstract

Breast cancer with multiple organ metastases sometimes shows a discordant response to treatment depending on the site of the lesion. In addition, oncologists generally convert the treatment upon progression of a small part of metastatic lesions in a specific organ, even if most metastatic lesions continue to respond well to the treatment. A 49-year-old postmenopausal woman had undergone mastectomy and sentinel node biopsy followed by breast reconstruction using a silicone implant for her left breast cancer 11 years before and was referred to our hospital due to the muscle weakness of her left upper arm. The patient had presumed multiple bone metastases, supraclavicular lymph node metastasis, and liver metastasis on images. Based on the judgment of estrogen receptor (ER)-positive and human epidermal growth factor receptor 2 (HER2)-negative metastatic breast cancer, the patient received fulvestrant, palbociclib, and denosumab, leading to the alleviation of upper arm muscle weakness. Positron emission tomography (PET) showed a decrease in fluorodeoxyglucose (FDG) uptake in the extrahepatic lesions and an increase in FDG uptake in the liver lesion at six months, and continued decrease of FDG uptake in the extrahepatic lesions and retained increase of FDG in the liver metastasis at 14 months. The patient, therefore, received additive alternate-day S-1 therapy to the first-line endocrine-based therapy, leading to the normalization of the elevated CA15-3 level, regression of the liver metastasis with decreased FDG uptake, and no additive adverse events. It was noteworthy that the addition of alternate-day S-1 therapy to the first-line endocrine-based therapy, clinically effective for extrahepatic lesions, led to the shrinkage of liver metastases without notable side effects.

## Introduction

The primary treatment goals for metastatic breast cancer are symptom palliation and prolongation of overall survival [1]. Breast cancer patients with estrogen receptor (ER)-positive, human epidermal growth factor receptor 2 (HER2)-negative, and non-life threatening metastasis, therefore, are generally treated with endocrine therapy, aiming not for cure of the disease but for maintaining their quality of life. However, disease progression of the metastatic lesion(s) with first-line endocrine therapy naturally leads oncologists to convert endocrine therapy to chemotherapy.

Physicians easily determine metastatic breast cancer as progressive disease when metastatic lesions are located in a single organ and show enlargement with the therapy. Some metastatic breast cancer, however, can show the situation that the vast majority of metastatic foci show favorable response to the first-line endocrine therapy, whereas a very small number of lesions in a different organ show disease progression [2]. This situation encompasses cases where their lesions initially shrink with the treatment followed by subsequent enlargement, as well as cases where they enlarge without responding to the treatment at all. Many cases of the former are referred to as “oligoprogression” and mainly result from the acquisition of resistance to the treatment [3]. On the other hand, many cases of the latter are attributable to the heterogeneity of metastatic lesions. Regardless of the mechanism underlying the disease progression, however, oncologists often face therapeutic dilemmas in treating such metastatic breast cancer, whether they should convert the therapy to another one or not.

The advent of taxanes has markedly changed the treatment strategy for liver metastases of breast cancer [4]. In short, conventional anticancer drugs hardly regressed the liver metastasis of breast cancer, whereas taxanes can often regress it. Physicians, therefore, sometimes encounter the situation where soft tissue and/or bone metastatic foci show favorable response to the treatment while liver metastasis progresses with it. In such situations, oncologists have difficulty in selecting the second-line therapy, especially when metastatic foci are detected after long term disease-free intervals, and all extrahepatic metastatic foci remain well-regressed with the first-line endocrine therapy.

Oral 5-fluorouracil (5-FU) analogues, such as capecitabine and S-1, have long been used for the treatment of metastatic breast cancer. These oral anticancer drugs generally have milder side effects than intravenous anticancer drugs. These oral agents, however, are rarely selected as the first- or second-line therapy for metastatic breast cancer. However, non-inferiority in efficacy against metastatic breast cancer has been demonstrated for S-1 in a comparative study with taxanes [5]. Furthermore, it has been reported that, in the treatment of gastrointestinal cancer, S-1 therapy on an alternate-day basis is superior to that on the standard dosing, offering equivalent efficacy with fewer side effects [6,7].

We herein report a case of breast cancer with multiple organ metastases, which responded well to endocrine-based therapy except for liver metastasis, followed by a favorable response of the liver metastasis to the additive alternate-day S-1 therapy on its progression [8].

## Case presentation

A 49-year-old postmenopausal woman was referred to our neurology department due to muscle weakness of the left upper arm. The patient had undergone mastectomy and sentinel node biopsy for her left ER-positive and HER2-negative breast cancer (pStage 2A), followed by breast reconstruction using a silicone implant 11 years before. The patient had received neither chemotherapy nor radiotherapy, but had received tamoxifen for five years and a luteinizing hormone-releasing hormone agonist for two years as adjuvant therapy, and had finished 10-year follow-up without any recurrences. Magnetic resonance imaging (MRI), performed to investigate the cause of upper arm muscle weakness, showed no spinal cord compression or narrowing of the intervertebral foramina but suggested possible cervical and thoracic spine metastasis from breast cancer (Figure 1).

**Figure 1:**
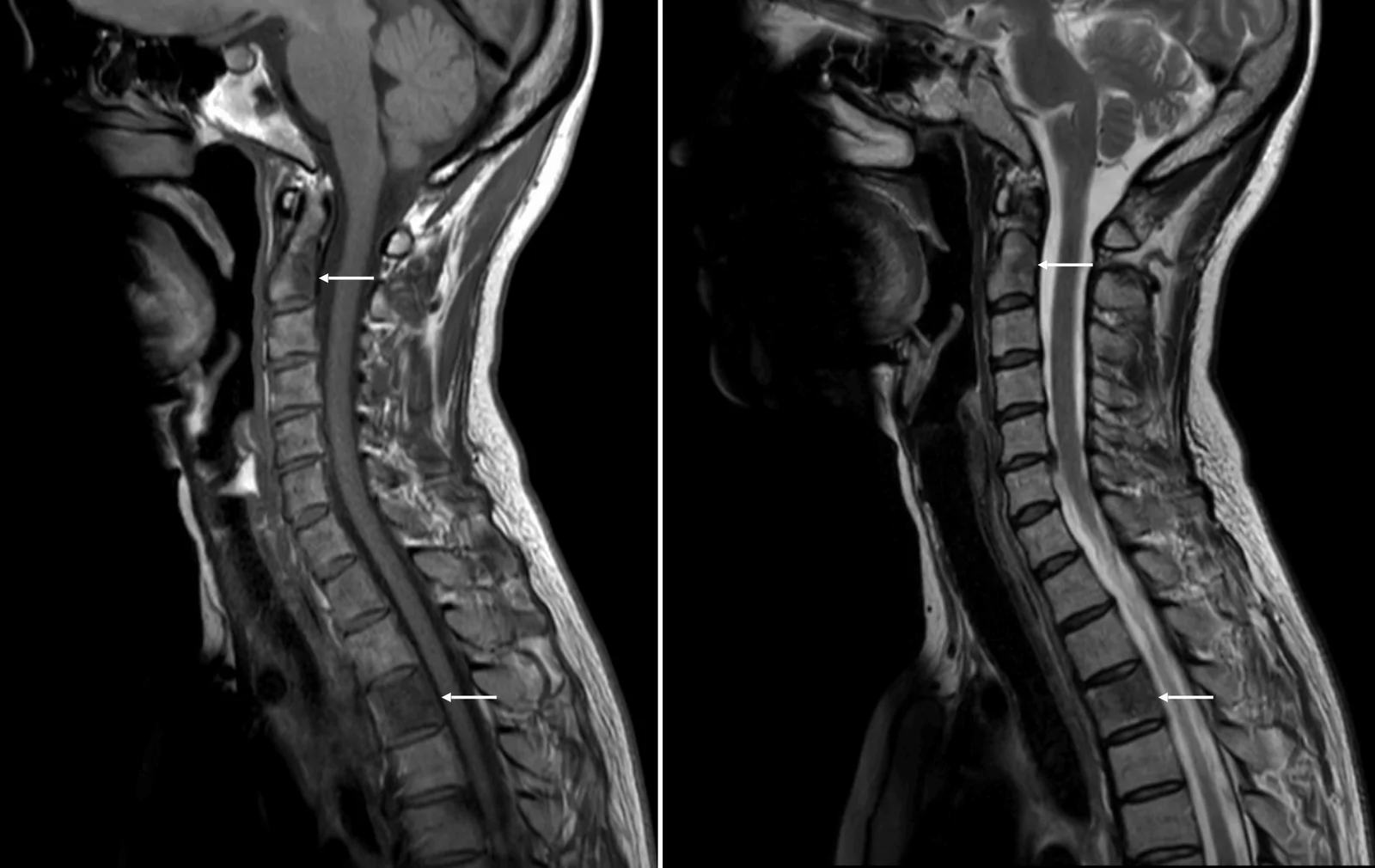
Cervical and thoracic spine magnetic resonance imaging (MRI) findings A. MRI of the C2 and Th3 spines showed hypointense on T1-weighted images (arrows). B. Low signals even on T2-weighted images (arrows) suggested either sclerotic changes of the cervical and thoracic spines or metastases of breast cancer with abundant fibrous components.

Palpation revealed an induration in the left supraclavicular region, which ultrasound highly suggested lymph node recurrence. Positron emission tomography/computed tomography (PET/CT) showed multiple bone metastases, including cervical and thoracic spine lesions, left supraclavicular lymph node metastasis, and liver metastasis (Figure 2).

**Figure 2:**
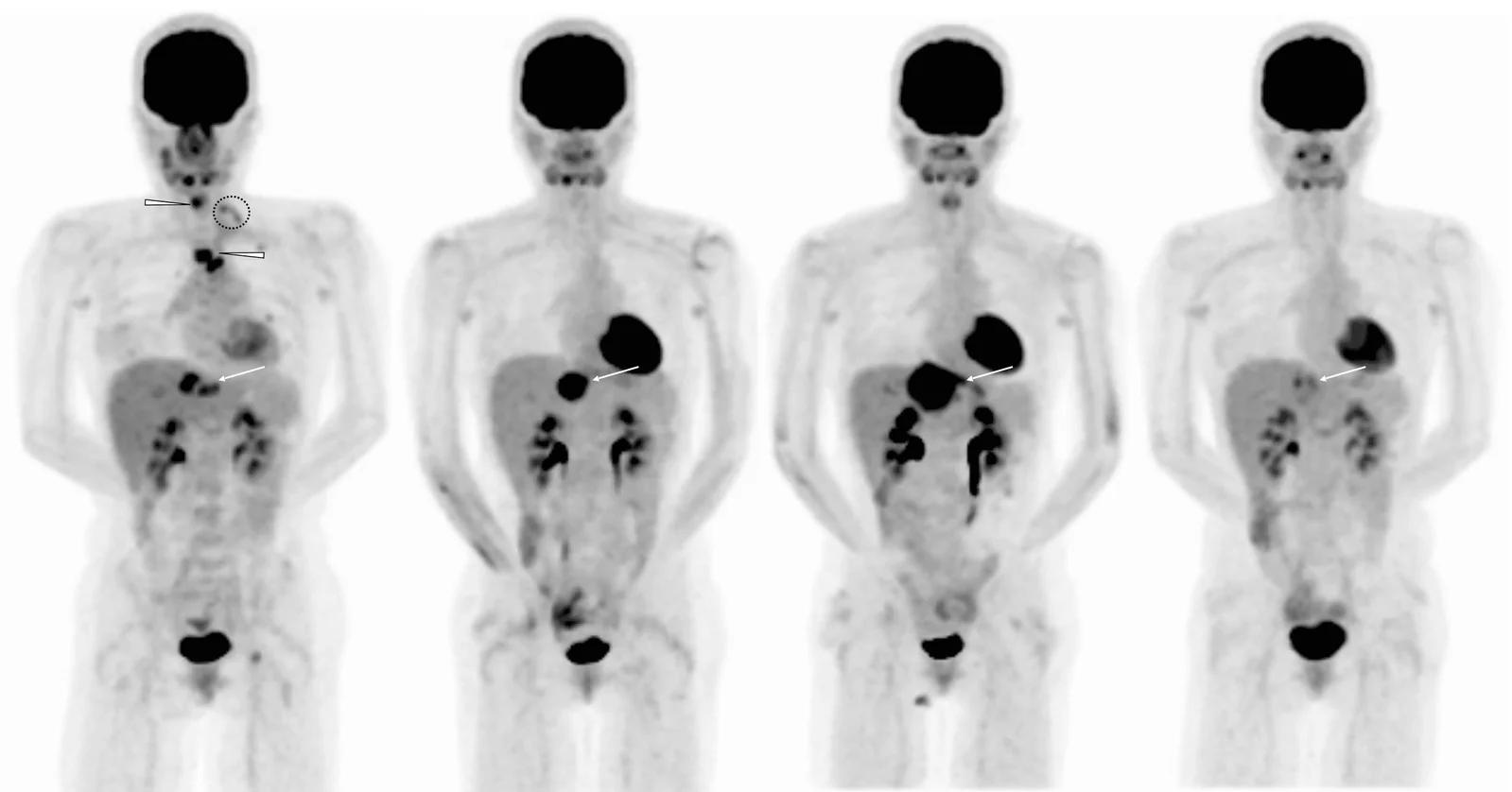
Positron emission tomography/computed tomography (PET/CT) findings A. PET showed fluorodeoxyglucose (FDG) uptake in multiple bones (arrowheads), supraclavicular lymph nodes (dotted circle), and the liver (arrow) before treatment. The presumed liver metastasis had a maximal standardized uptake value (SUVmax) of 8.72. B. PET showed a decrease in FDG uptake in the extrahepatic lesions and a marked increase in FDG uptake in the liver (arrow) six months after the initiation of endocrine-based therapy. The presumed liver metastasis had a maximal standardized uptake value (SUVmax) of 12.13. C. PET showed continued decrease of FDG uptake in the extrahepatic lesions and the retained increase of FDG uptake in the liver metastasis (arrow) at 14 months after the start of endocrine-based therapy. The presumed liver metastasis had a maximal standardized uptake value (SUVmax) of 12.93. D.PET showed continued decrease in FDG uptake in the extrahepatic lesions and a marked decrease of FDG uptake (arrow) in the liver metastasis at six months, i.e., total 21 months on treatment, after the addition of alternate-day S-1 therapy. The presumed liver metastasis had a maximal standardized uptake value (SUV max) of 5.21.

Based on the above findings, the neurologist concluded that the upper-limb muscle weakness was attributable to the supraclavicular lymph node metastasis. However, our explanation that it might cause possible further worsening, albeit infrequently, of the upper arm symptoms due to the hematoma formation made the patient prefer not to undergo core needle biopsy of the supraclavicular lymph node. Similarly, the patient did not undergo liver biopsy due to concerns about the risk of liver hemorrhage associated with needle biopsy. The patient, therefore, received fulvestrant, palbociclib, and denosumab based on the judgment of ER-positive and HER2-negative metastatic breast cancer, leading to the improvement of muscle weakness in two months. Ultrasound also showed marked regression of the metastatic supraclavicular lymph node (Figure 3).

**Figure 3:**
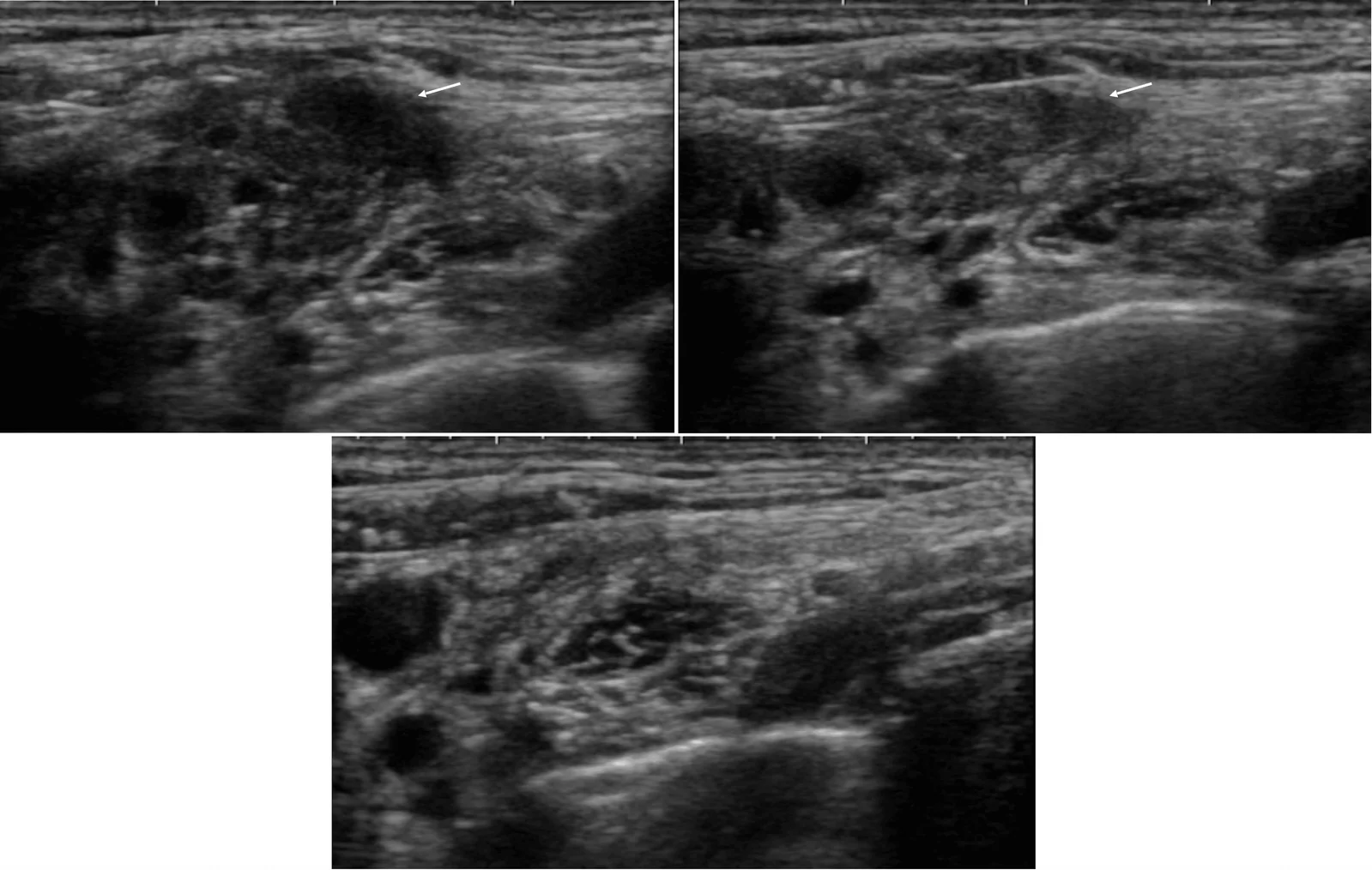
Ultrasound findings of the supraclavicular lymph node A. Ultrasound showed an ill-defined supraclavicular metastatic lymph node (arrows) before starting the endocrine-based therapy. B. Ultrasound showed marked shrinkage of the supraclavicular metastatic lymph node (arrows) in two months with the first line endocrine-based therapy. C. Ultrasound showed continued marked shrinkage of the metastatic lymph node with internal high echoes (arrows) in 11 months after the start of therapy, i.e., at the time of initiation of the S1 therapy.

PET/CT showed decreased fluorodeoxyglucose (FDG) uptake in the bone and lymph node lesions and increased FDG uptake in the liver lesion at six months, and continued decrease of FDG uptake in the extrahepatic lesions and retained increase of FDG uptake in the liver lesion at 14 months (Figure 2). These findings led us to judge that while the treatment with fulvestrant, palbociclib, and denosumab effectively controlled all extrahepatic lesions, it had limited or no efficacy against the liver metastasis. We once considered the use of stereotactic body radiotherapy for the liver metastasis, but opted against it because the lesion was large, measuring approximately 6 cm. In addition, we considered switching the therapy to a different endocrine therapy, but judged that a treatment acting not through the ER was preferable for the liver metastasis. We further judged that the first-line endocrine-based therapy was still effective for extrahepatic lesions. We, therefore, fully explained to the patients our assessment of the first-line treatment efficacy and the main side effects associated with each regimen considered for the second-line therapy. In the discussion of the second-line treatment, the patient had a strong preference for the treatment that did not cause hair loss, making us select oral 5FU analogues. Differences in the side effect profiles of capecitabine and S-1 led the patient to choose S-1. In addition, the explanation that an alternate-day dosing regimen often results in milder side effects than the standard S-1 regimen led patients to choose the alternate-day schedule. The patient, therefore, received additive alternate-day S-1 therapy to the first-line endocrine-based therapy. The additive alternate-day S-1 therapy reduced the CA15-3 levels, having increased beyond normal levels from two months before the initiation of S-1 therapy, within two months and normalized them in and after three months (Figure 4).

**Figure 4:**
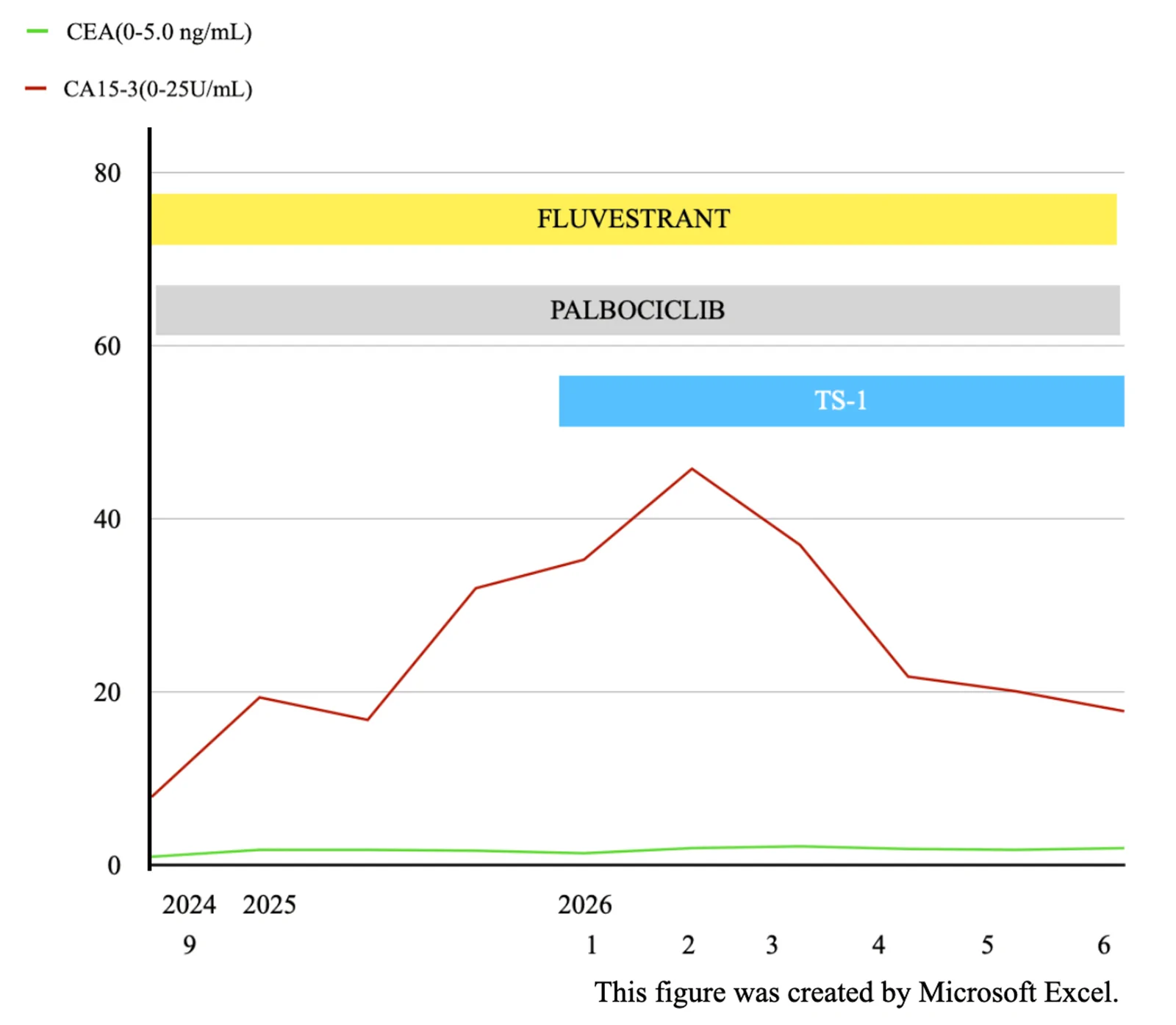
Serial changes of treatment regimens and tumor marker levels Serum CA15-3 levels elevated synchronously with the increase of fluodeoxyglucose uptake in the liver metastasis on positron emission tomography, but decreased shortly after the addition of the alternate-day S-1 therapy.

In addition, ultrasound showed marked shrinkage of the liver metastasis (Figure 5).

**Figure 5:**
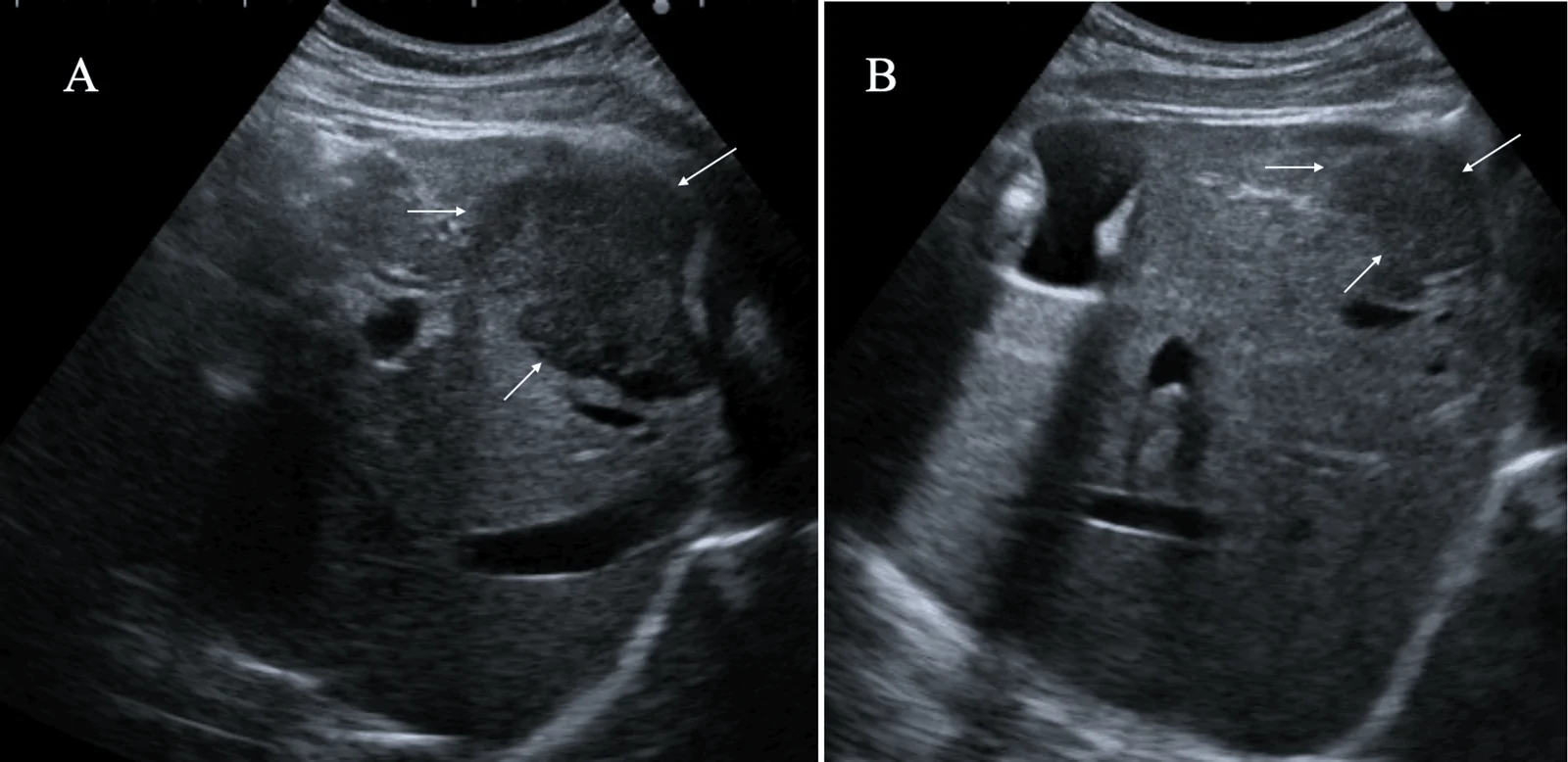
Ultrasound findings of the liver metastasis A. Ultrasound showed enlargement, 5.9 cm × 4.3 cm, of the liver metastasis (arrows) before the addition of the alternate-day S-1 therapy. B. Ultrasound showed marked shrinkage, 3.2 cm × 1.5 cm, of the liver metastasis (arrows) after the addition of the alternate-day S-1 therapy.

The patient had no new adverse events, including blood tests, after the initiation of S-1 therapy. PET/CT six months after the initiation of S-1 therapy showed a marked decrease in the maximal standardized uptake value, i.e., from 12.93 to 5.21, in the liver lesion (Figure 2). The patient has been receiving the additive S-1 therapy for seven months and is scheduled to continue the chemo-endocrine therapy as long as the favorable response continues.

## Discussion

It is well known that patients with liver metastasis of breast cancer had extremely dismal clinical outcomes, especially in the era before the advent of taxanes [9]. Oncologists, therefore, often experienced in the treatment of metastatic breast cancer that extrahepatic metastatic lesions showed a favorable response to some kind of anticancer therapy, whereas liver metastasis progressed with the same therapy. Because oligoprogression itself occurred very infrequently in the pre-taxane era, the discordance of clinical response between different metastatic sites did not pose a major clinical problem.

Taxanes naturally play the most important role in the treatment of liver metastasis of breast cancer [4,10]. Taxane-based chemotherapy, therefore, was an important therapeutic option when judged only by the aspect of therapeutic efficacy also in this case. It, however, seemed reasonable that much less toxic anticancer agents than taxanes were more suitable for this case, at least as first-line chemotherapy, due to both the very long disease-free interval, i.e., more than 10 years, and incurable disease status. Although we did not perform a biopsy of the liver metastasis, we considered the possibility that the liver metastases could be ER-negative, well explaining the lack of response to the first-line endocrine-based therapy, and therefore decided to add chemotherapy to the first-line therapy for better response. Thus, the addition of a less toxic chemotherapeutic agent, i.e., possible long-term administration, to the first-line endocrine-based therapy, having been definitely effective for the extrahepatic lesions, seemed to be a more practical option in this case.

Oncologists often use oral anticancer drugs, such as capecitabine and S-1, that allow long-term treatment for metastatic breast cancer. Capecitabine, a prodrug of 5-FU, is used globally as an anticancer drug with much less toxicity compared to that of various intravenous anticancer drugs. Treatment with capecitabine, however, often has to be interrupted by the capecitabine-induced unique side effect of hand-foot syndrome [11]. On the contrary, S-1 is a drug consisting of three components, such as tegafur, i.e., a prodrug of 5-FU, gimeracil that inhibits the catabolism of 5-FU, and oteracil potassium that prevents gastrointestinal toxicity, leading to higher serum 5-FU concentration than that of oral 5-FU / capecitabine and therefore low compliance either with a four-weeks-on/two-weeks-off or with a two-weeks-on/one-week-off regimen [12]. By contrast, it is well known that the alternate-day dosing regimen of S-1 results in marked improvement in medication compliance [6,7]. In fact, the addition of S-1 to the first-line endocrine-based therapy has caused no notable side effects, including on blood tests in this case. We had planned to switch the therapy from the additive alternate-day S-1 therapy to taxane-based chemotherapy when confirming no response of the liver metastasis to the second-line therapy. This patient, however, fortunately got a decrease in tumor marker levels and shrinkage of the liver metastasis shortly after the addition of alternate-day S-1 therapy to the endocrine-based therapy. We cannot even speculate whether S-1 monotherapy was effective for the liver metastasis in this case. In addition, it was also unclear whether S-1 therapy could control the extrahepatic lesions as effectively as the first-line endocrine-based therapy. The addition of alternate-day S-1 therapy, therefore, seemed a rational approach in this case. However, had we performed a needle biopsy of the liver metastasis, we might have been able to demonstrate the rationality of the treatment strategy employed in this case.

## Conclusions

Physicians sometimes observe that recurrent ER-positive and HER2-negative breast cancer after long disease-free intervals responds discordantly to first-line endocrine-based therapy, depending on the sites of recurrence. They generally switch treatment to taxane-based chemotherapy when liver metastasis progresses, even if all extrahepatic lesions continue to respond well to the first-line endocrine-based therapy. It was noteworthy that the addition of alternate-day S-1 therapy to the first-line endocrine-based therapy, clinically effective for extrahepatic lesions, led to the shrinkage of liver metastases without notable side effects.
